# A week long “pep” talk – initial and 2–3-year longitudinal data on the Ottawa Psychiatry Enrichment Program (OPEP)

**DOI:** 10.1186/s12909-022-03216-x

**Published:** 2022-03-09

**Authors:** Elliott Kyung Lee, Alexandra Morra, Khalid Bazaid, Abdellah Bezzahou, Kevin Simas, Christopher Taplin, Soojin Chun, Jess G. Fiedorowicz, Alan Bruce Douglass

**Affiliations:** 1 The Department of Psychiatry, Faculty of Medicine, University of Ottawa, Ottawa, ON Canada; 2 University of Toronto, Toronto, ON Canada; 3 Carleton University, Ottawa, ON Canada

**Keywords:** Attitudes, Recruitment, Career choice, Enrichment program, Medical students, Psychiatry

## Abstract

**Background:**

Recruitment to psychiatry as a career has been challenging in Canada and abroad despite the known shortage and increasing burden of psychiatric issues globally. Deterrents to choosing psychiatry as a career include its negative stigma and paucity of knowledge about the field. The study goal was to evaluate the Ottawa Psychiatry Enrichment Program (OPEP), a one-week extracurricular program about psychiatry as a career for 1st and 2nd year medical students. We hypothesized OPEP would improve students’ attitudes towards psychiatry, and positive changes would be sustained 2–3 years later following their residency match. We hypothesized there would be a high recruitment of OPEP attendees to psychiatry programs.

**Methods:**

1st and 2nd year medical students from Canada applied to OPEP. Attendees completed the Attitudes Towards Psychiatry Questionnaire (ATP-30) at three times: before OPEP (PreOPEP), after OPEP (PostOPEP) and after their Canadian Residency Matching Service (CaRMs) match 2–3 years later. OPEP ATP-30 scores were compared to third-year student ATP-30 scores before and after their psychiatry rotation. Data were analysed using Friedman non-parametric ANOVA and post hoc testing by either Wilcoxon rank sum test, Wilcoxon matched pairs signed rank test, or parametric Welch independent t-test as appropriate. Effect sizes of group mean differences were calculated using Cohen’s “d”.

**Results:**

Between 2017–2018, 29/53 Canadian applicants were selected for OPEP. 100%, 93.1% and 75.8% of OPEP students completed the PreOPEP, PostOPEP, and CaRMs ATP-30 surveys respectively. 43% of OPEP attendees matched to psychiatry. PostOPEP ATP-30 scores (mean = 133, median = 137, SD = 10.6) were significantly higher than PreOPEP ATP-30 (mean score = 121, median = 122, SD = 9.3, *p* < 0.001) and CaRMS ATP-30 (mean = 126, median = 127, SD = 12.3, *p* < 0.02) scores. OPEP effect size on ATP-30 scores was large (d = 1.2) but decreased 2–3 years later (*p* = 0.078, d = 0.44). 97/202 students completed the ATP-30 before and after their psychiatry rotation (clerkship). Clerkship effect size on improvement in ATP-30 was moderate (d = 0.39). There was a non-significant difference between OPEP CaRMS ATP-30 and post clerkship ATP-30 scores (median 127 vs 121, *p* = 0.056).

**Conclusions:**

OPEP ameliorated attitudes toward Psychiatry, but improvement deteriorated longitudinally. Strategies for program design, and innovations to boost/retain improvements during clerkship years are discussed.

**Supplementary Information:**

The online version contains supplementary material available at 10.1186/s12909-022-03216-x.

## Background

Globally, 45% of the world’s population live in a country without the recommended ratio of 1 psychiatrist per 100,000 people [1]. In Canada, an estimated 1 in 5 individuals will develop a mental illness [2]. Despite these alarming numbers, patients in Canada are often waiting 6 months to 1 year for therapy, highlighting the severe shortage and need for more psychiatrists to bridge this gap in care provision [3]. Further exacerbating this gap is that psychiatrists in Canada are the lowest paid specialty among medical specialties in Canada and in the bottom quartile in the United States [4, 5]. Favorable features of psychiatry as a career have included its emphasis on neuroscience, diagnostic complexity, and favorable job opportunities, while significant deterrents, have included stigma of psychiatry to medicine colleagues as well as lack of exposure in medical school [6]. Manassis et al. highlighted the most influential factors influencing psychiatry residents towards their residency were initial interest, clerkship experiences, and participation in enrichment activities [7]. To begin a career in psychiatry, medical students in Canada must first match to a psychiatry residency program thru the Canadian Residency Matching service (CaRMS), where students must apply upon graduation to a discipline(s) and program(s) of interest in the country. CaRMS then provides an objective and transparent application and matching service for medical training throughout Canada, as programs evaluate candidates after completing an assessment process and vice versa. Recruitment to psychiatry residency programs among medical students in Canada has historically been stable at 4–6% according to CaRMS data, which fails to meet the chronic community need [8]. Despite the significant need for psychiatrists, for the last 20 years up until 2020, every iteration of CaRMS has produced unmatched psychiatry positions in Canada in the first round [9]. In an effort to improve recruitment to psychiatry residency programs, since 2017, several Canadian schools have initiated “enrichment” programs” to enhance student interest in psychiatry [8]. These extracurricular programs, typically of 3–5 days duration, have invited medical students to learn more about psychiatry as a profession early in their medical school careers, to improve attitudes towards psychiatry and advance interest in the field as a career. Several studies have demonstrated that a more positive attitude towards psychiatry is associated with an increased likelihood of pursuing this as a career [10, 11].

Previous reviews of enrichment initiatives suggest they help with improving attitudes and interest, but there is a paucity of longitudinal data on efficacy for maintaining improved attitudes and increasing recruitment [12, 13]. To date, only 3 programs have longitudinal data on efficacy. The Combined Accelerated Program in Psychiatry (CAPP) introduced in Maryland in the 1970s showed that 70% of their students who ranked psychiatry first in their introductory year went on to train as psychiatrists in residency [14]. In Western Australia, the Claasen program introduced students to psychiatry in 2008 through a week-long session of morning seminars and afternoon community hospital and mental health visits. Over a 6 year follow up, 17/47 (36%) students who were followed were either interested in a psychiatry career, or definitely considering this [15]. The Psychiatry Institute for Medical Students, based in Toronto, Canada, was another week-long program aimed at improving recruitment. This program succeeded in recruiting 76/178 (43%) participants from 1994–2005 to psychiatry residency programs [16]. The Psychiatry Early Experience Program (PEEP), a more intense program developed in Kings College in London, England, paired students in their first year with trainee psychiatrists and had them visit 2 days every 6 months throughout the 5 years of students’ medical training [17]. Although the investigators were unable to determine whether their learners ended up becoming psychiatrists, they did show that after a 3 year follow up, improved positive attitudes towards psychiatry were sustained among 22/40 respondents.

The purpose of our study was to evaluate the efficacy of an Ottawa enrichment program in improving the attitudes of Canadian medical students towards psychiatry. We hypothesized that students selected to our enrichment program would have more positive attitudes towards psychiatry compared to students just starting clerkship, and that the OPEP program could further enhance this interest. We speculated that improvements in attitudes to psychiatry would be greater than improvements seen by the clerkship experience at the University of Ottawa, and that such interest could be sustained over medical school leading to increased recruitment to psychiatry residency programs.

## Methods

The Ottawa Psychiatry Enrichment Program (OPEP) was initiated in 2017 to 1^st^ and 2^nd^ year students; those from Ottawa as well as across Canada were invited to apply. Ottawa applicants were recruited through announcements in the preclerkship psychiatry block in the curriculum, as well as through the local psychiatry mental health interest group. Program information was also disseminated nationally through the Canadian Organization of Undergraduate Psychiatric Educators (COUPE) to attract applicants from across Canada. Applicants submitted a 300-word paragraph and optional materials if they felt these would be helpful. Submissions were evaluated and ranked by a blinded panel of 2–3 staff doctors and 1–2 learners (resident, medical student) based on 1) perceived interest in psychiatry as a field (more heavily weighted) and 2) potential for future clinical and/or academic excellence. The panel was asked to rank the top 10 applicants from Ottawa, and the top 10 from outside Ottawa each year, to potentially offer the opportunity to participate in OPEP. Depending on the year and resources, between 12–16 positions were filled. Approximately 60% of applicants were selected from Ottawa.

Mirroring the Psychiatry Institute of Toronto, OPEP began with morning sessions consisting of interactive seminars given by instructors with reputations for academic excellence and/or excellent student evaluations. Afternoons consisted of observerships where learners were paired one-to-one with clinical supervisors to see real patients together and experience different areas of psychiatry. The hope was to destigmatize psychiatry for attendees, and for them to see role models in psychiatry, and be able to appreciate the work life balance psychiatry affords as a rewarding career as well as the impact of the field on the patients they serve. For details of the structure of the OPEP week, see Table 1.

**Table 1:** A sample calendar outlining the one-week schedule of the OPEP program. Morning blocks consisted of 1-h interactive lectures featuring different areas of psychiatry. Instructors were asked to primarily discuss their particular practice and inspiration for psychiatry, though some introduction of didactic material was encouraged. Instructors were primarily paid through departmental funds from each respective hospital, while residents were reimbursed through their resident education fund. One lecture was a client panel where patients spoke of their experiences in the mental health system. Instructors were from mixed backgrounds, with many notable senior instructors having a track record of academic excellence including national and international prizes, along with younger instructors early in their careers with strong teaching evaluations to provide a role model and a varied perspective for students to visualize a potential career in psychiatry. Recruitment of instructors deliberately reflected at least 50% female instructors, with particular attention being paid to recruit female leaders in our department, including our residency training director, and the chair of our department among others. The Department of Psychiatry of Ottawa provided lunch each day with selected residents with a history of academic excellence and noted interest in education, in randomized clusters of approximately 3–4 attendees per resident. Lunch provided an opportunity to have an open time to network and discuss psychiatry as a career in a relaxed setting, without attending staff doctors present. Afternoons consisted of 2 h “observerships where students were matched with preceptors to get more “hands on” experience in different areas of psychiatry (mostly in outpatient settings if possible). Each afternoon, learners were matched with different psychiatrists, to provide the widest breadth of experience of psychiatry as a field. Examples of different observerships included forensic psychiatry, child psychiatry, geriatric psychiatry, sleep medicine and many others

Time	Monday	Tuesday	Wednesday	Thursday	Friday
0800–0900	BREAKFAST	BREAKFAST	BREAKFAST	BREAKFAST	BREAKFAST
0900–1000	Lecture	Lecture	Lecture	Lecture	Lecture
1000–1100	Lecture	Lecture	Lecture	Lecture	Lecture
1100–1200	Lecture	Lecture	Resident Panel	Lecture	Lecture
1200–1300	LUNCH	LUNCH	LUNCH	LUNCH	LUNCH
1300–1400	Travel	Travel	Travel	Travel	Travel
1400–1600	Clinical observation	Clinical Observation	Clinical Observation	Clinical Observation	Clinical Observation

The Attitudes Towards Psychiatry (ATP-30) survey was used to assess students’ sentiments towards mental health and psychiatry as a career. The ATP-30 is a 30 item 5-point Likert-based scale developed by Burra et al. with the intention of encapsulating the attitudes of medical students towards psychiatry [18]. The scoring ranges from 30 – 150, with a score of 90 and above considered indicative of a positive attitude towards psychiatry [11]. Sample questions are added as Appendix 1. Its psychometric properties demonstrate strong test–retest reliability and a Cronbach’s alpha of 0.83 [18]. As depicted in a systematic review by Wei et al., the ATP-30 is the only scale measuring healthcare professionals’ perceptions towards psychiatry that was created in a Canadian context and specifically tailored towards medical students and residents [19]. Written informed consent was obtained and then students completed the ATP-30 survey prior to OPEP (“PreOPEP”), after OPEP (“PostOPEP”), and then again after they completed the Canadian Residency Matching Program (CaRMS) match 2–3 years later. At this time, students were contacted by e-mail, and in addition to completing the ATP-30 survey again, they also indicated to what residency program they matched using SurveyMonkey. The Institute for Mental Health Research (IMHR) Research Ethics board approved all experimental methods and protocols for this study.

**Fig. 1:**
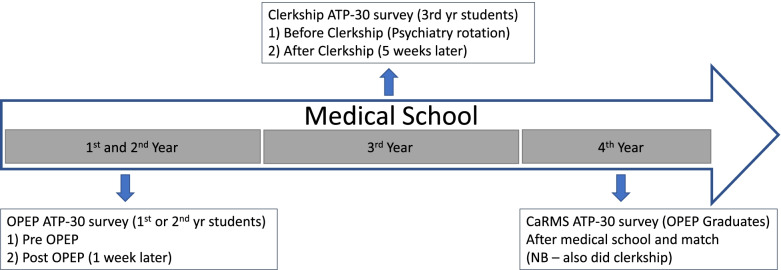
This figure illustrates the timeline of medical school, and when students in each arm of the study received the Attitudes Towards Psychiatry (ATP-30) survey during their medical school experience. OPEP students (bottom) received the ATP-30 survey at 3 time points, including before and after OPEP which took place at the end of their 1^st^ or 2^nd^ year, and again at 4^th^ year, after they were aware of their Canadian Residency Matching Service (CaRMS) results. Clerkship students (top) completed the ATP-30 survey just before their psychiatry rotation and 5 weeks later after completion (placed within their 3^rd^ year of medical school)

Two other groups were available for study: the ATP-30 was also administered to the entire 2020 University of Ottawa psychiatry clerkship cohort as well as part of the 2021 cohort, prior to the start of clerkship, as well as right after clerkship 5 weeks later. These students provided informed consent. This latter sample was part of a separate project that has since been published [20] and was incorporated into this analysis to serve as a comparison to the OPEP students. The University of Ottawa Office of Research and Integrity provided ethics approval and approved all experimental methods for use of these data. ATP-30 survey results from students who had already completed the OPEP program (*n* = 6) were excluded from these clerkship data. Figure1 illustrates the temporal sequence of available ATP-30 data. All datasets used and/or analysed during the current study are available from the corresponding author on reasonable request.

### Statistical design

Since questionnaires were completed anonymously, all data were aggregated by grouping (PreOPEP, PostOPEP, CaRMS, BeforeClerkship, AfterClerskhip) and analysed as unmatched data. In order to decide on the use of parametric or non-parametric tests, ATP-30 data from each of the 5 groups noted above were assessed for normality of distribution using the Shapiro–Wilk test; this showed that two groups differed significantly from normality: 1) the PostOPEP group (mean = 133, median = 137, interquartile range = 14.5, Shapiro–Wilk *p* = 0.007); and 2) the BeforeClerkship group (mean = 117, median = 115, interquartile range = 13, Shapiro–Wilk *p* = 0.012). Therefore, to be conservative, non-parametric group comparisons were used throughout. All 5 groups were compared simultaneously using the Friedman non-parametric ANOVA, with post-hoc testing by either the Wilcoxon rank-sum test with continuity correction (non-parametric independent t-test), or the Wilcoxon matched-pairs signed-rank test with continuity correction (non-parametric paired t-test), as appropriate. The required significance level was increased from *p* = 0.05 to account for multiple comparisons, by means of the Holm-Bonferroni method [21]. Effect sizes of group mean differences were calculated using Cohen’s “d” with acknowledgement that the ATP-30 distribution only approximated normality in the PreOPEP and AfterClerkship group. All analyses were done with the ‘R’ statistical software, version 6.3.1 [22].

## Results

Between 2017–2018, 24/313 (7.7%) first- and second-year medical students from the University of Ottawa applied to OPEP from the 2019 and 2020 graduating classes. Applicants also came from the 2021 class but have not been followed as of yet. 29 additional students applied from across Canada, for 29 total positions (13 in 2017, 16 in 2018). Seven students were offered positions but declined, either being unable to come due to travel, competing obligations, or not responding. Two of these students came the following year. This final OPEP group consisted of 19 female and 10 male students. Many positive comments were made on exit evaluations. Students voiced enjoying hearing about choosing psychiatry as a career and learning about subspecialties. Networking with other peers from across the country over a shared passion for psychiatry, as well as having observerships with a 1:1 ratio between staff and student were also other identified positive attributes. See Table 2 for thematic analysis of comments made regarding the OPEP program.

**Table 2:** Summary of Thematic Analysis Evaluation Questionnaire completed by OPEP participants following the OPEP program Response rate = 29/29 participants (100%). All evaluations were completed anonymously. Each question had space for a numerical score, and invitation to write comments for specific day etc. Sample questions in the evaluations are included below the table

THEME	SAMPLE COMMENTS
Showed variety of areas of psychiatry	“Gave a great snapshot of different areas of psychiatry” “Great that we get to see different areas of psychiatry” “Excellent experience; loved the opportunity to see diversity in field” “Participating in OPEP was very enlightening and changed some of my previous thoughts regarding what a career in psychiatry would be like.”
High quality of teachers	“Favorite part of OPEP was that preceptors were great at teaching and made for a pleasant experience” “Dr. X was an excellent mentor and encouraged me to ask questions and peruse my interests in psychosis” “Fascinating to hear clinical stories. All speakers were clearly passionate about their field.”
Organization & Logistics	“Very well organized” “Ensure physicians have afternoons with patients”
Diversity of Speakers	“Resident panel was most likely the most useful since it is the stage we are closest at.” “Loved having the opportunity to chat with residents in a casual way during lunch.” “Client stories were great; loved the patient panel”
Content of Program	“Add more basic content to teach other skills in psychiatry.” “It may be good to have a psychiatrist who has focus on post partum depression, perinatal issues, eating disorders; [this] is an area I would have been interested to learn more about.” “Would be great to see ECT conducted” “Would have been good to learn more about the residency program, and matching to residency programs”

1. On a scale of 1–5, 5 being excellent and 1 being extremely poor, please rate:

a) Setting (program organization, orientation, met objectives, met expectations, comfort)

b) Food (rated for each day of the program)

c) Lectures (rated for each day of the program, separated by lecturer)

d) Observership (rated for each day)

2. What did we do that was RIGHT? (open ended question)

3. What do we need to IMPROVE and how can we do that? (open ended question)

**Fig. 2:**
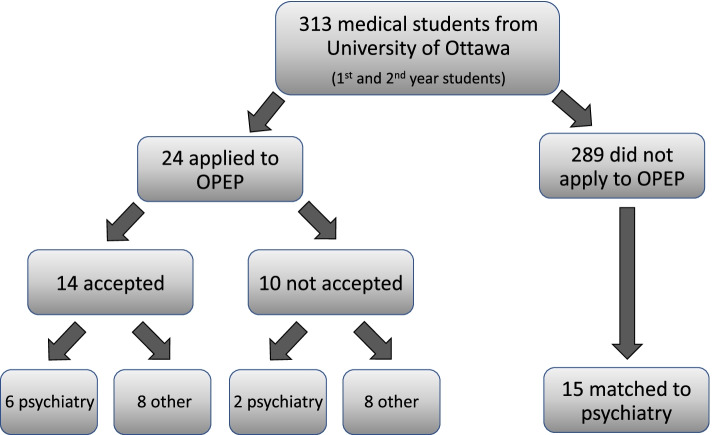
The following figure depicts the path that 2019 and 2020 University of Ottawa medical students followed upon graduation. Students are categorized to those who applied to OPEP, who were further categorized to those accepted versus those that were not accepted. Additionally, students who did not apply were also followed (far right rectangles). Subsequent follow up revealed how many students in each group matched to psychiatry upon graduation (bottom rectangles)

All 29 OPEP students completed the ATP-30 survey prior to OPEP starting (“PreOPEP scores”); 27 students (93%) filled out the ATP-30 upon OPEP completion (“PostOPEP scores”). 23/29 (79%) students subsequently have graduated and entered the CaRMS match (6 students graduate in 2021). 22/23 (96%) of ATP-30 surveys were collected after the CaRMS match (“CaRMS”). Among these students, 10/23 (43%) students matched to psychiatry, 8 matched to family medicine, and remaining graduates matched to other specialties, and one is unknown. Among the 24 University of Ottawa students who applied to the program from the 2019 and 2020 graduating classes, 14 were accepted to OPEP, and 6/14 (43%) students matched to psychiatry. Among the 10 students who were declined acceptance from these classes, 2/10 (20%) matched to psychiatry programs. 15/289 (5%) of University of Ottawa students from the classes of 2019 and 2020 who did not apply to OPEP matched to psychiatry (see Fig*. *2).

**Fig. 3:**
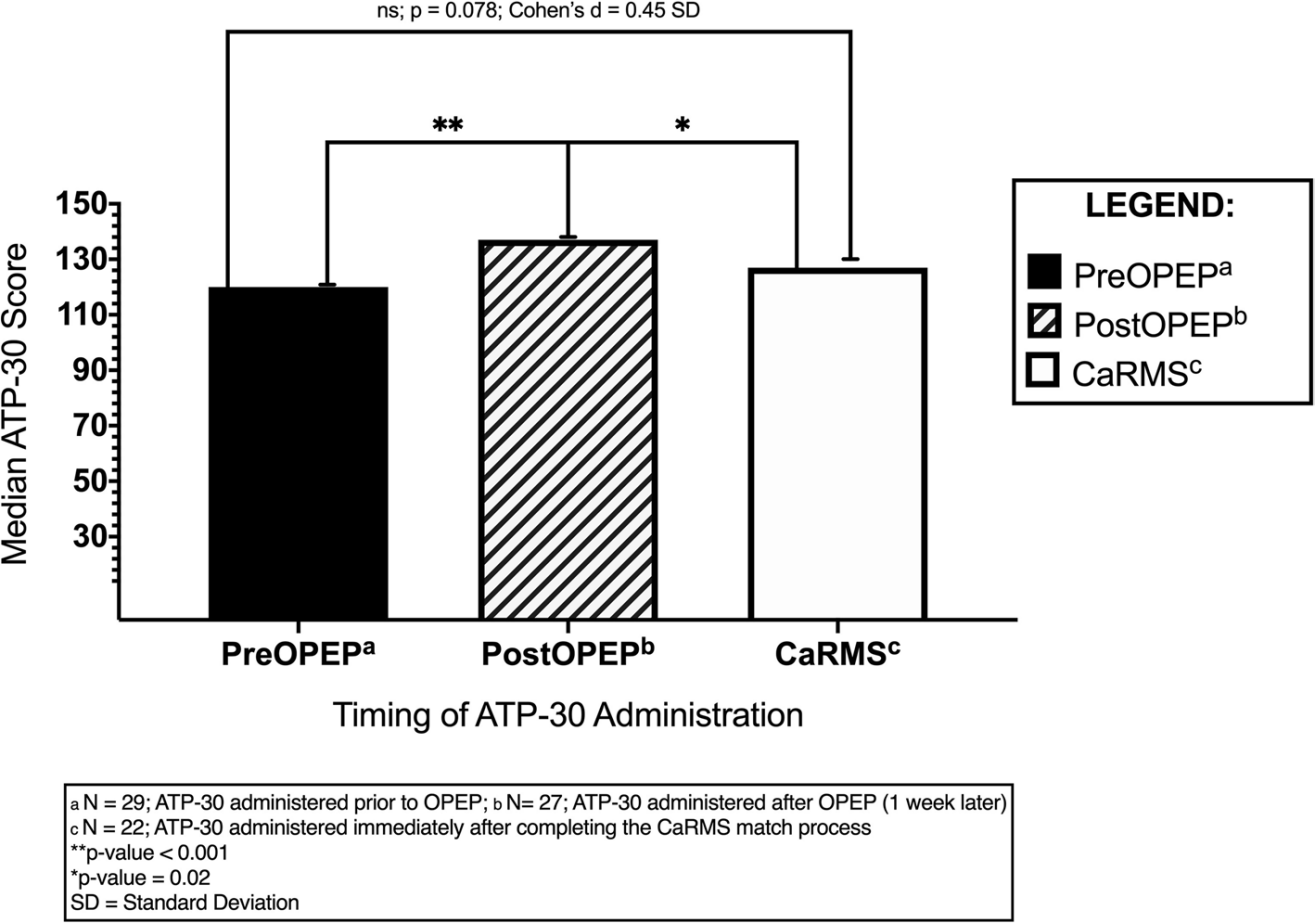
This figure represents the median ATP-30 scores of students **a**) prior to starting OPEP (*n* = 29), and **b**) after completing the OPEP program (*n* = 27) one week later, as well as 2–3 years later after they completed the Canadian Residency Matching Service (CaRMS) match (*n* = 22)

Friedman ANOVA conducted on the ATP-30 median scores showed highly significant differences between the 5 groups (p-value < 0.001). Post-hoc pair-wise testing of group medians was performed as follows: (PreOPEP, PostOPEP), (PostOPEP, CaRMS), and (PreOPEP, CaRMS) using Wilcoxon rank-sum tests. PreOPEP ATP-30 scores for attendees were high, as expected (mean score = 121, median = 122, SD = 9.3). Following the completion of OPEP, PostOPEP median scores (mean = 133, median = 137, SD = 10.6) were significantly higher than PreOPEP scores (mean = 121, median = 122, Cohen's d = 1.2 SD, *p* < 0.001). While ATP-30 scores declined 2–3 years afterwards following CaRMS (mean = 126, median = 127, SD = 12.3), they still showed a moderate effect size improvement compared to PreOPEP ATP scores (Cohen’s d = 0.45 SD, *p* = 0.078). The PostOPEP median was also significantly greater than the CaRMS median score, though to a lesser degree (137 vs. 127, *p* = 0.02). Median PreOPEP ATP-30 scores were non-significantly lower compared to the median CaRMS ATP-30 scores (122 vs. 127, *p* = 0.078). See Fig. 3*.*

**Fig. 4:**
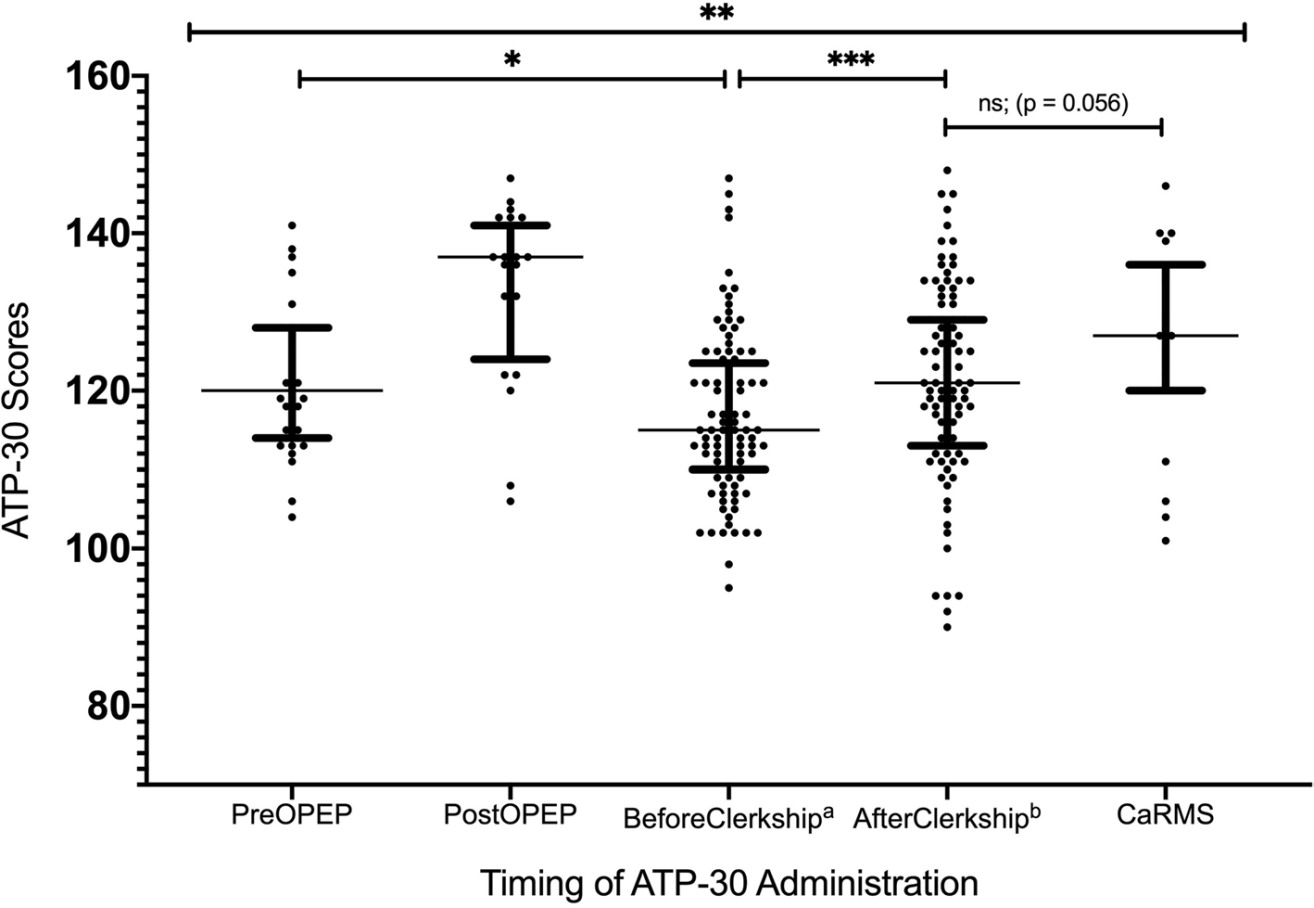
This figure depicts the Attitudes Towards Psychiatry (ATP-30) scores for the respective student cohorts. Each dot represents an individual student’s ATP-30 score, with the horizontal line representing each cohort’s median, and the horizontal bars depicting the 25th and 75th percentile (interquartile range, IQR). ^a^*N* = 97; ATP-30 was administered prior to starting core psychiatry clerkship rotation. ^b^*N* = 97; ATP-30 was administered after 5 weeks of core psychiatry clerkship rotation. *p-value = 0.018; This was determined via Wilcoxon non-parametric independent t-test. **p-value < 0.001; This was calculated using Friedman non-parametric ANOVA. ***p-value < 0.001; This was determined via Wilcoxon non-parametric paired t-test

Comparisons were also done between the 3 OPEP groups and the contrast group that only attended their compulsory psychiatry clerkship. A post-hoc Wilcoxon independent t-test showed a significant difference between PreOPEP ATP-30 scores and BeforeClerkship ATP-30 scores (medians 122 vs 115, *p* = 0.018). A similar test showed only a non-significant result towards a difference between CaRMS and AfterClerkship ATP-30 scores (median 127 vs 121, *p* = 0.056). A Wilcoxon paired t-test showed a significant improvement from BeforeClerkship to AfterClerkship (median 115 to 121, *p* < 0.001) with a moderate effect size (Cohen’s d = 0.39). See Fig. 4*.*

## Discussion

These data suggest that over the course of the OPEP experience, attitudes to psychiatry improved considerably, even in a group already favorably disposed. Presumably the large effect size occurred not only as a result of the high-quality instructors to whom students were exposed, but possibly also because of the exposure to others with a shared interest, possibly giving a sense of community by being around like-minded individuals. These improvements regressed 2–3 years later, though still tended to be higher than scores prior to starting OPEP and scores of non-OPEP students who had finished clerkship. In addition to adding the longitudinal outcome of participation in an enrichment program which few previous studies have followed, these data build on these by illustrating the results of selecting participants who already have a high interest early in their career, as opposed to selecting those who might be more undecided. Furthermore, unlike previous evaluations of other enrichment programs, these data also highlight the outcomes of those who were not selected for the program (either because they applied and were not selected, or did not apply). To our knowledge, this is the only study of enrichment programs that has tracked this kind of data. About 5% of learners from Ottawa who did not apply to OPEP still went on to match to psychiatry, comparable to historical match rates from CaRMS [9]. Moreover, unlike other research into such enrichment programs, comparison can be made to changes in attitudes of OPEP participants to a standard group of clerkship students, and illustrates that even with a predetermined high interest, further improvements in attitudes towards psychiatry could be seen with OPEP (although waned with time), that were beyond those improvements seen with a “standard” clerkship experience for those presumed to have less predetermined interest.

These data suggest that the selection process indeed selected candidates for OPEP who had a highly favorable attitude towards psychiatry, as expected. While OPEP was designed to further improve attitudes towards psychiatry, it is also possible that a social desirability bias contributed to the significant increase in ATP-30 scores after OPEP. Despite being reassured of the survey’s anonymity, students may have thought that reporting more ‘positive’ attitudes toward psychiatry made them a more ‘desirable’ candidate for residency. Clinical clerkship has been shown to improve attitudes towards psychiatry among medical students [23] and was also seen in these data. Previous work, however, has identified decay in attitudes towards psychiatry during the latter part of medical school post clerkship [24–26], and was also seen with these OPEP graduates in the CaRMS match.

Almost half (43%) of the Ottawa OPEP graduates went on to match to psychiatry two to three years later. These results are comparable with results from learners enrolled in other enrichment initiatives such as the Claasen institute (36%) and the Psychiatry Institute from Toronto (43%) [15, 16]. Among 23 total students that matched to psychiatry from this 2-year cohort of 313 Ottawa students, 15/23 students (65%) never applied to OPEP. These data suggest that for 8/23 students, interest in psychiatry occurred earlier hence their interest in applying to OPEP, whereas for the other 15 students, possibly, their interest developed later in their medical careers. However, it is unclear whether these students may have attended another enrichment program such as the Psychiatry Institute of Toronto (the only other program advertising nationally to students). Additionally, it is possible that some Ottawa students did not apply because of OPEP’s novelty, as they might not have known enough about OPEP or possibly were uncertain about its credentials. Furthermore, it is possible that such students were not interested or had competing obligations. Further work could also be considered to enhance selection procedures, since only one third of the Ottawa students who matched to psychiatry were identified through OPEP.

The strengths of our study include the inclusive nature of our longitudinal follow up with a high (96%) response rate after the CaRMS match, and the availability of comparison data with a clerkship class. Unfortunately, however, just under half the clerkship classes completed the ATP-30 before and after clerkship, possibly introducing bias.

Limitations of this study include the small sample size. Consequently, caution is warranted in interpreting these results though recruitment results to residency programs are consistent with other enrichment programs and highly significant. Additionally, it is not known if students who did not match to psychiatry possibly ranked psychiatry highly but were not selected. Anecdotally, one OPEP graduate mentioned ranking 3 psychiatry programs before ultimately getting matched to her 4^th^ choice in family medicine. As an additional observation, among the 13 students who came from outside of Ottawa to OPEP and matched, none matched to psychiatry in Ottawa. These data consequently suggest that enrichment programs might have more success in attracting internal candidates to internal programs, more so than bringing external candidates to such a program, though numbers are small so caution in interpretation is warranted. Further longitudinal follow-up would be ideal, as 6 students matched in 2019, while 17 matched in 2020; this may increase bias because the majority of candidates followed matched in one year. It is also unclear whether these students who were not followed up would be significantly different from the students who did complete follow up questionnaires. It would be ideal to have had all the clerkship students fill out the ATP after matching in CaRMS as well, but unfortunately this was not feasible. Such results could help determine the extent to which attitudes might be predicted to decay after clerkship and may help understand whether enrichment programs like OPEP might “raise the floor” on such patterns of deterioration in attitudes in students endorsing early career interest. The 2021 CaRMS match, however, is different than any other match in previous memory due to the impact of COVID-19 (e.g., no national electives).

Psychiatry as a field has become more popular as measured by recent increases in students matching to psychiatry residency programs in the US and Canada [9, 27]. In Canada the percentage of medical students matching to psychiatry was 7.5% in 2020 [9]. 2020 was the first year where 100% of the psychiatry positions across Canada were filled by the 1^st^ iteration. One possible contributing factor is the emergence of several enrichment programs. Since 2017–8, UBC, McMaster, and Western had also started their own enrichment programs, though they were primarily open only to local medical students [8]. Together with Ottawa and Toronto, this represents 5/17 (29%) of medical schools in Canada. In the future, it may also be helpful to offer such programs for the francophone schools in Canada. Such programs could be viewed as not only increasing but also possibly retaining interest in psychiatry, as students applying already identify early interest. It may also behoove departments to examine deterrents to psychiatry, since these and other data suggest deterioration of attitude and interest over 2–3 years. The improvements seen with these data may also be helpful for educators to consider, as participation in such a program consumes significant human resources in terms of time commitment. An enrichment program is most likely to be effective with quality lecturers and supervisors. Such teachers may be more inclined to participate knowing that their efforts in such a program do change attitudes of those involved, and high-quality instructors and mentors have been highlighted to be helpful towards recruitment [10, 28].

Psychiatry, more than other specialties, is prone to stigma, both from patients and our colleagues [29], which threatens recruitment. As we accelerate further into a virtual education world, the incorporation of video conferencing software may render benefit in future OPEP or similar enrichment sessions. Moreover, the Ontario Telehealth Network (OTN), a clinical provincially funded telehealth portal, is widely accessible in Ottawa, and could be used for the observership component of OPEP. Pre-recorded sessions could be available to students after OPEP as well, potentially bolstering its capacity for longitudinal impact. Reminder or “anniversary” emails, akin to similar notifications on social media, may additionally serve as a means of refreshing students’ memories of the OPEP experience, to possibly invigorate steps towards interest in psychiatry. More robust programs such as the PEEP program from the UK offer a glimpse to the possibilities of sustaining improvements in attitudes towards psychiatry with continuous contact with preceptors, but some schools may not have such resources to dedicate to learners. Perhaps some kind of middle ground with an enrichment program added to the aforementioned considerations could be more widely feasible and part of a broader strategy for increasing interest in psychiatry both as a field of medicine and potentially as a career for a larger number of medical school programs. Overall, long term implementation of enrichment programs may be helpful to expose students early in their career to role models in psychiatry, and possibly with further measures might promote development of student/mentor relationships that have been suggested to be helpful for recruitment [25]. Given the climate of COVID, and the difficulty of students travelling for external electives, we urge educators in psychiatry to consider more innovative approaches to help improve recruitment to the field.

## Conclusions

When a psychiatry department considers different recruitment strategies, one imperative is to assess whether an enrichment program has potential to a) improve attitudes towards psychiatry and b) lead to meaningful long-term differences in learners choosing to pursue psychiatry as a career (i.e. residency program). Furthermore, when considering program development, it is unclear if it is more useful to focus on active recruitment of ambivalent candidates versus retention of existing students with a keen interest. That is to say, is it better to choose participants who are “on the fence” to evaluate if such a program assists with encouraging the pursuit of psychiatry (a “recruitment” strategy), or does it render benefit to select those already indicating a high interest, as a means to solidify and galvanize such interest (a “retention” strategy)? Walaszek opined in a 2017 commentary that “identifying medical students with pre-existing interest in psychiatry and fostering that interest may be particularly effective [at recruiting] to psychiatry as a career” [30]. These data highlight the longitudinal outcome of a “retention” approach with such a program to addressing the shortage of psychiatrists in medicine. An early enrichment program for medical students can successfully identify early interest in psychiatry as a career and can significantly improve attitudes towards psychiatry in such learners. Our data suggest that there is value in improving these attitudes early on but there can be deterioration with time. Since the selection criteria for the OPEP program missed two thirds of the learners who eventually would match to psychiatry in Ottawa, future exploration of other factors in clerkship and beyond with possibly more of a “recruitment” approach could be fruitful to identify and cultivate further interest in psychiatry among more senior medical students. For those students who had completed OPEP, since their attitudes are likely to deteriorate by the time they declare their career interests, future work is needed to develop innovations to sustain initial improvements of such a program and identify factors leading to their deterioration in attitudes to psychiatry.

## Supplementary Information

**Additional file1.** Sample items from the ATP-30 Questionnaire[1] (first 5 items).

## Data Availability

All datasets used and/or analysed during the current study available from the corresponding author on reasonable request. The raw data are not fully anonymized and consequently, are not available publicly, but analysis of data in aggregate in this study is anonymized.
