# Supplementary material for: A week long “pep” talk – initial and 2–3-year longitudinal data on the Ottawa Psychiatry Enrichment Program (OPEP)
Source: BMC Med Educ. 2022 Mar 9;22:163. doi: 10.1186/s12909-022-03216-x (PMC8906362; doi:10.1186/s12909-022-03216-x)
Supplement: Supplementary file 1 — Additional file1. Sample items from the ATP-30 Questionnaire[1] (first 5 items). [file 12909_2022_3216_MOESM1_ESM.docx]

Appendix 1:

Sample items from the ATP-30 Questionnaire^1^ (first 5 items), answers A-E, and scored accordingly, from:

1. Strongly Agree
2. Agree
3. Neutral (no opinion)
4. Disagree
5. Strongly Disagree

1. Psychiatry is unappealing because it makes so little use of medical training.

2. Psychiatrists talk a lot but do very little.

3. Psychiatric hospitals are little more than prisons.

4. I would like to be a psychiatrist.

5. It is quite easy for me to accept the efficacy of psychotherapy.

1. Burra P, Kalin R, Leichner P, et al. The ATP 30-a scale for measuring medical students' attitudes to psychiatry. *Med Educ.* 1982;16(1):31-38.
